# Late widespread skeletal metastases from myxoid liposarcoma detected by MRI only

**DOI:** 10.1186/1477-7819-6-62

**Published:** 2008-06-18

**Authors:** Sammy A Hanna, Yassar A Qureshi, Lee Bayliss, Lee A David, Paul O'Donnell, Ian R Judson, Timothy WR Briggs

**Affiliations:** 1 London Bone and Soft Tissue Tumour Service, Royal National Orthopaedic Hospital, Stanmore, HA7 4LP, UK; 2 Sarcoma Unit, Royal Marsden Hospital, London, SW3 6JJ, UK

## Abstract

**Background:**

Myxoid liposarcoma is the second most commonly occurring sub-type of liposarcomas. In contrast to other soft tissue sarcomas, it is known to have a tendency to spread toward extrapulmonary sites, such as soft tissues, retroperitoneum, and the peritoneal surface. Bony spread, however, is not as common.

**Case presentation:**

We report an unusual case of diffuse skeletal metastases from myxoid liposarcoma occurring 13 years after treatment of the primary tumour in the left lower limb. The skeletal spread of the disease was demonstrated on MRI only after other imaging modalities (plain radiography, CT and TC99 bone scans) had failed to detect these metastases.

**Conclusion:**

MRI is an extremely sensitive and specific screening tool in the detection of skeletal involvement in these types of sarcomas, and therefore, should be a part of the staging process.

## Background

Liposarcomas are a group of soft tissue malignancies in which the direction of differentiation is toward fatty tissue. The trunk and the lower extremities are the most likely sites of tumour development [1,2]. Liposarcomas are classified histologically into three grades (Table 1), with metastases occurring according to this grade. Low-grade lesions account for <10%, intermediate grade 10% to 15%, and high grade > 50% [2-4]. Myxoid liposarcoma (intermediate-grade) is the second most frequently occurring subtype, accounting for one third of cases, and representing about 10% of all adult soft tissue sarcomas [5]. It is well known the majority of soft tissue sarcomas metastasise to the lungs [3,4]. MLS, in contrast, has a different pattern of metastatic spread. It has a tendency toward extrapulmonary sites, such as soft tissues, retroperitoneum, peritoneal surface, and the axilla. However, bony spread is not as common [1-3]. We report a case of MLS associated with unusual late diffuse skeletal metastases detected only by MR imaging.

**Table 1:** Classification of Liposarcomas

Low Grade	Intermediate Grade	High Grade
Well differentiated Lipoma like Sclerosing Inflammatory	Myxoid	Dedifferentiated Round cell Pleomorphic

## Case presentation

**Figure 1:**
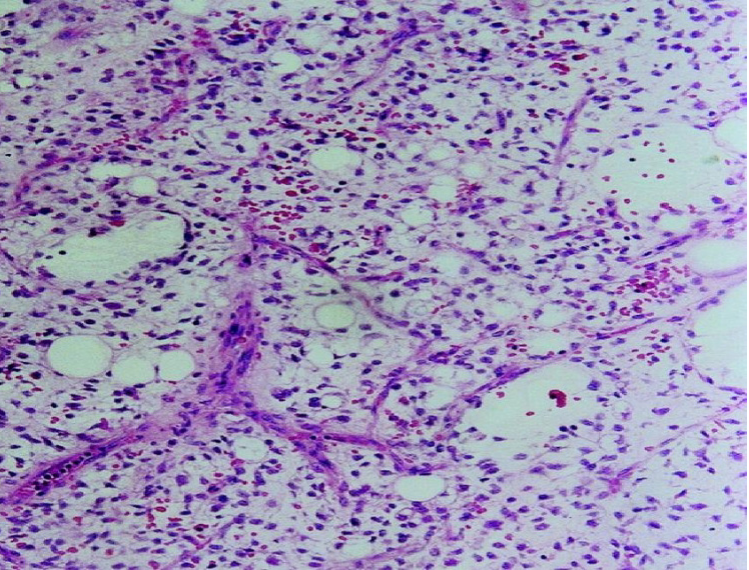
Histology slide of myxoid liposarcoma showing lipoblasts and capillaries in a predominantly myxoid stroma (H&E stain, 10× magnifications).

**Figure 2:**
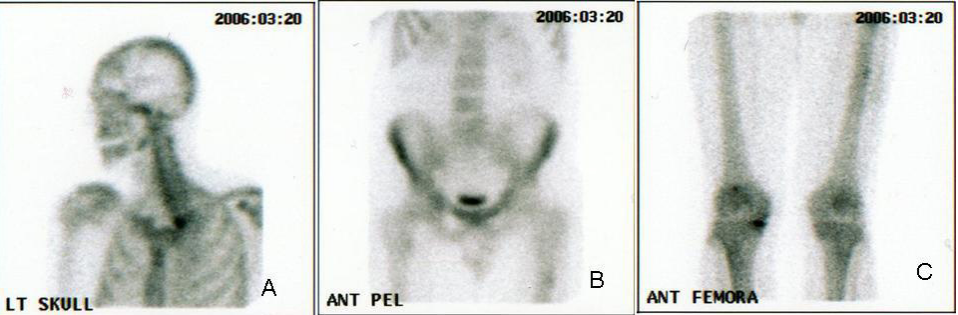
Technetium bone scan. No significant uptake in the skull (a), spine and pelvis (b), and proximal femora (c).

**Figure 3:**
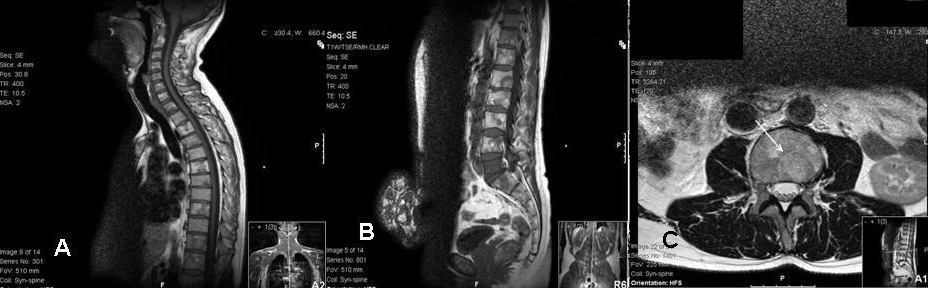
Diffuse abnormal bone marrow signal throughout the spine. Note multiple lesions with high-signal centres on T1W, reflecting the myxoid/fatty nature of the deposits. (a) Sagittal T1W MR iamge of cervical and thoracic spines. (b) Sagittal T1W MR iamge of lumbosacral spine. (c) Transverse section T2W MR image in L3.

A 51-year old Caucasian male presented to his local hospital with a history of an enlarging painful mass in his left thigh in 1992. A needle biopsy was in keeping with the diagnosis of a myxoid liposarcoma, and full staging imaging did not reveal any secondary lesions. A wide local excision was performed and subsequent histopathological examination showed a completely excised high-grade myxoid liposarcoma. Fractions of radiotherapy (60 Gy) were administered postoperatively. In both 1997 and 1999, local recurrences occurred, for which surgical excisions with wide margins were achieved. Surgery was supplemented with radiotherapy on both occasions. In 2004, the tumour disseminated to three distant sites including the liver, chest wall, and neck. The metastases were successfully resected. Histopathological examination revealed similar cellular findings do the primary tumour (Figure 1). As the surgical margins were clear, no further treatments were deemed necessary at that stage. In 2005, the patient started complaining of back pain but plain spinal radiographs, CT (chest, abdomen and pelvis) and a TC 99m bone scan (Figure 2) did not reveal any signs of spinal disease. However, a spinal MRI (Figure 3) showed a diffuse and widespread abnormal bone marrow signal not detected previously. A bone marrow aspirate suggested the diagnosis of metastatic disease. During 2006, chemotherapy (Doxorubicin, Ifosfamide, and Trabectedin) and radiotherapy (fractions of 20 Gy) were administered, resulting in good symptomatic recovery, with no disease progression. In 2007, the patient sustained a pathological fracture in his left proximal femur following a fall. An MRI revealed multiple well-defined foci of abnormal signal throughout both femora and in the right pubis and both ischia, in addition to the fracture. He subsequently underwent a proximal femoral replacement at our institution with the view of achieving good pain relief and a reasonable functional outcome. Histopathological examination revealed extensive bone necrosis secondary to radiotherapy, but no viable tumour cells were identified. Single agent Trabectedin was given postoperatively. The patient died of his disease eleven months later, 15 years after initial diagnosis.

## Discussion

Our case demonstrates the possibility of diffuse bone involvement with myxoid liposarcoma, even late in the course of the disease. This uncommon pattern of spread should certainly be taken into account when staging patients and determining their prognosis. It also shows the value of MRI in the detection of these metastases. Other methods for assessing skeletal metastases, including plain radiography, CT scanning and Tc 99m isotope scanning, would appear on the evidence of this, and other cases [6-9], to be insensitive to myxoid liposarcoma. Schwab *et al*. [8] have shown that even PET scans lack sufficient sensitivity to detect spinal involvement in this disease and recommended the use of total spine MRI when screening for metastases in MLS patients. Because bone secondaries are not common in soft tissue sarcomas, skeletal staging would not be routinely performed in many centres. Furthermore, relying solely on CT as a staging tool in these cancers might result in a significant number of metastases being missed. MRI, on the other hand, is an extremely sensitive tool to bone marrow replacement by abnormal tissue, as the high contrast between fat (marrow) and water (tumour) demonstrates metastatic lesions at an early stage [10]. We believe that whole-body MRI may be a more appropriate staging investigation of this particular tumour with unique clinical features. Because our patient was initially asymptomatic, there was no way to detect the metastatic bone lesions and no reason to suspect they were present, and this may explain the late diagnosis. We do not know when skeletal lesions first occurred, as no spinal MRI was obtained between initial diagnosis and 2005. In addition, all other surveillance imaging investigations were negative. *Ishii et al*. [6] have reported two cases of relatively early bone metastases (2 and 4 years latency) not detected by bone scans. They attributed the normal accumulation of radiotracers to the likely diminished metabolic bone activity. Other authors have suggested that the myxoid stroma may prevent labelled glucose from reaching cells in sufficient quantity to be detected by the scanner [8].

## Conclusion

By reporting this case, we emphasise two important points; diffuse skeletal metastases can occur late in the course of MLS (13 years latency in this case), and MRI appears to be more sensitive than any other imaging modality in detecting bone marrow involvement in this disease. It is therefore essential to consider the value of whole-body MRI in the management of patients with intermediate (myxoid) to high grade liposarcomas. We recommend this be used in the initial staging process, and whenever local recurrence/metastases are suspected.

## Competing interests

The authors declare that they have no competing interests.

## Authors' contributions

SA, YQ, and LB Performed the literature search and drafted the manuscript. LD Contributed to the discussion and the conclusion. PO, IJ, and TWB: Critically reviewed and improved the manuscript.
